# American Dental Association

**Published:** 1890-10

**Authors:** 


### EDITORIAL.

### American Dental Association.

The thirtieth annual meeting of this body was held at Excelsior Springs, Mo., August 5th, and the three succeeding days. It was a successful meeting in almost every respect. The attendance, though not so large as sometimes, was very good indeed. Some of the faces that have been familiar heretofore, were absent of course, but there were many new ones, and it is always pleasant to make new acquaintances as well as to meet old ones.

Much good work was done by the body. Many excellent papers were read and discussed. We shall hope to have a good report of these in the October No. of the REGISTER.

A very important matter that received the attention of the society was the appointment of a committee of five, in compliance with a request from the Southern Dental Association to cooperate with a committee of like number appointed by the latter body, who, together, should take into consideration the propriety of holding a World's Dental Meeting at the time of the Columbian Fair in Chicago, in 1893. The association concurred unanimously in the request and appointed the committee.

These committees met for consultation and effected the organization; to it reference will be made hereafter.

The place of holding the next annual meeting was not fixed, but was left in the hands of the Executive Committee. Excelsior Springs was a very pleasant place for holding the meeting. Its accommodations were ample, and every thing was done for the comfort and pleasure of the guests, and all left, feeling pleased that they had been there.

